# Comparative hemolymph proteomic analyses of the freezing and resistance-freezing *Ostrinia furnacalis* (Guenée)

**DOI:** 10.1038/s41598-024-52792-z

**Published:** 2024-01-31

**Authors:** Zhu-Ting Zhang, Huan Wang, Hui Dong, Bin Cong

**Affiliations:** 1 Shenyang Agricultural University, Shenyang, 110866 Liaoning People’s Republic of China; 2 Kaili University, 556011 Kaili, People’s Republic of China

**Keywords:** Biological techniques, Chemical biology, Climate sciences, Environmental sciences

## Abstract

The Asian corn borer, *Ostrinia furnacalis* (Guenée) (Lepidoptera: Crambidae), is one of the most harmful pests of maize in Asia. It poses a significant threat to maize production, causing economic losses due to its strong ecological adaptation. In this study, we compared and analyzed the hemolymph proteome between freezing and resistance-freezing *O. furnacalis* strains using two-dimensional gel electrophoresis to gain insights into the mechanisms of cold resistance. The results revealed that 300–400 hemolymph protein spots were common, with 24 spots showing differences between the two strains. Spectrometry analysis revealed 21 protein spots, including 17 upregulated spots and 4 downregulated ones. The expression of upregulation/downregulation proteins plays a crucial role in the metabolism, energy supply, and defense reaction of insects. Proteomics research not only provides a method for investigating protein expression patterns but also identifies numerous attractive candidates for further exploration.

## Introduction

The Asian corn borer (ACB), *Ostrinia furnacalis* (Guenée) (Lepidoptera: Crambidae), is one of the most harmful pests of maize in Asia^1,2^ and is distributed throughout India, Southeast Asia, China, Korea, Japan, Australia, New Guinea, Solomon Islands, and Western Micronesia^3,4^. ACB causes enormous damage to maize production with yield losses of 10 – 80% due to strong ecological adaptation^5^. As a poikilotherm, the migration and population dynamics of ACB have been altered due to climatic changes. Goto et al. (2001) suggested that ACB had a well-developed cold hardiness mechanism including a system for terminating diapause before winter and also a system for accumulating glycerol after diapause^6^. This system could help ACB overcome cold stress, which often resulted in changes in energy consumption, metabolic rate, development, and innate immunity^7^. The metabolic adaptations to cold stress involve the synthesis of responsive proteins. Through proteomic analysis, an investigation was conducted to better understand the molecular adaptation mechanisms of ACB to cold stress.

At present, protein analysis has regained importance because understanding the inner working of a cell requires delving into the study of cellular proteins themselves^8^. Proteomic studies are recognized as a pivotal scientific pursuit in the post-genomic era^9^. The gold standard for quantitative proteome analysis involves combining high-resolution protein separation (via isoelectric focusing / SDS-PAGE) in two-dimensional gel electrophoresis (2DE) with mass spectrometry (MS) or tandem MS (MS/MS) identification of selected protein spots. Significant technical advances related to 2DE and protein MS have increased the sensitivity, reproducibility, and throughput of proteome analysis while creating an integrated technology^10^. The entire protein complement expressed by a genome or by a cell or tissue type is called proteome. Interestingly, a disparity exists between the amount of mRNA in a cell and the proteins actually produced^11^. When analyzing tissue samples, the studies on mRNA and proteomics were performed. However, the analysis of body fluids (e.g., serum, urine, cerebrospinal fluid, and hemolymph) is restricted to proteomics because most proteins in these fluids are secreted from other tissues.

Insects possess a single extracellular fluid circulating throughout their body. In an open circulatory system, the “blood” flows freely within the body cavity (hemocoel) and directly contacts all internal tissues and organs. Therefore, a more accurate term for insect blood is hemolymph. Hemolymph serves as the primary location for most of the body water and acts as a reserve during dehydration. Insect hemolymph plays crucial roles in nutrient and hormone transport, amino acid storage, water balance, responses to injury, and immunity. Low temperatures can increase the viscosity of hemolymph, leading to a significant reduction in physiological transport^12^. In some cold-adapted insect species, the seasonal production of specialized ice-binding proteins contributes to cold-hardening^13^. These proteins are typically secreted into the hemolymph to regulate ice formation, enhancing cold hardiness by (a) preventing the growth of ice crystals to stabilize the supercooling point, (b) initiating ice formation to facilitate controlled ice crystal growth, and (c) inhibiting the recrystallization of ice crystals. Much of our understanding of the proteins involved in these functions has been achieved from studies of the Lepidoptera, with relatively large quantities of hemolymph available for biochemical analysis^14^. The ACB has an open circulatory system containing hemolymph, which surrounds the tissues of the insect with blood. Nutrients and oxygen are delivered to all body parts through the hemolymph, which is also an essential depository for nutrition and energy. Studies focus on this tissue because changes occur in the hemolymph due to climatic changes, which may affect the growth and behavior of the ACB. The variations in hemolymph content across different developmental stages or physiological conditions make the proteome maps of freezing and resistance-freezing ACB proteins the foundation for constructing a wealth of information about hemolymph physiology. Not only the comparative analysis of the proteins related to freezing but also the optimization of the whole technology concerning proteomic analysis of ACB contribute to future proteomic studies.

At present, ACB is the most economically important corn stalk borer in Asia. The environmental temperature is a key abiotic factor involved in the biocontrol of pests by fungi and bacteria. The immune response was stronger in the lipopolysaccharide (LPS)-treated group at 30 °C than in the treated group fat lower temperatures in *Tenebrio molitor* larvae^15^. The regulation of body temperature through certain behaviors in some insects contributed to their survival when infected with entomopathogen^16^. It is essential to understand the proteins that cause resistance-freezing so as to create new strategies or find alternative ways to control the pest.

## Results

### Protein map of hemolymph from resistance-freezing and non-resistance-freezing ACBs

**Figure 1:**
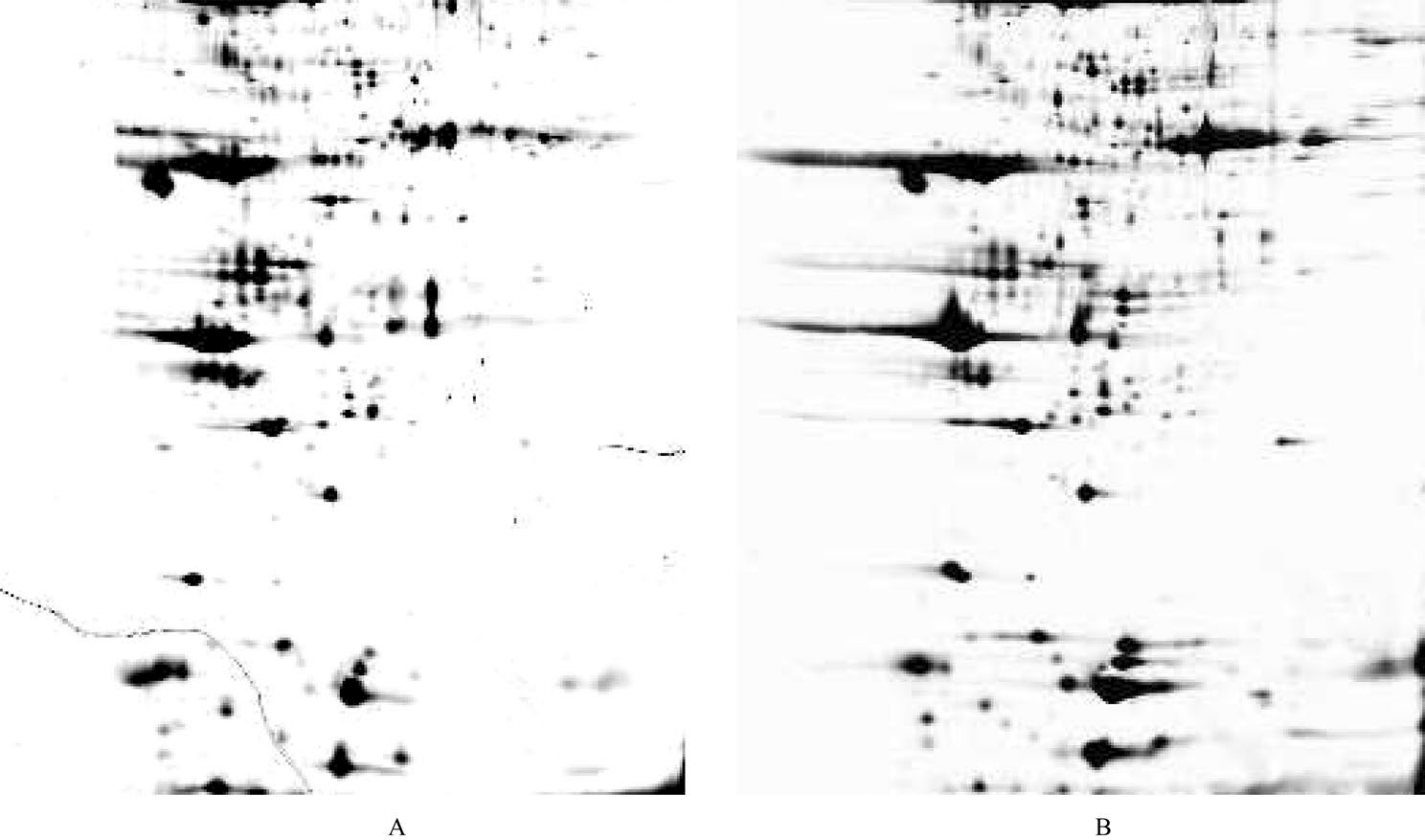
2D PAGE analysis of protein from resistance-freezing and non-resistance freezing ACB larvae. Proteins were separated according to isoelectric points on immobilized 3–10 pH gradients, and separated by 12.5% SDS-PAGE. The samples derive from the same experiment and that gels/blots were processed in parallel. A total of 400 spots were detected, and 21 spots were differentially expressed in resistance-freezing and non-resistance-freezing ACB larvae, respectively. (**A**) Plasma proteins of hemolymph from resistance-freezing ACB larvae. (**B**) Plasma proteins of hemolymph from non-resistance freezing ACB larvae.

The 2D standard pattern of hemolymph from resistance-freezing and non-resistance freezing ACB larvae is presented in Fig. 1. Overall, 300–400 protein spots were expressed in resistance-freezing/non-resistance-freezing ACB larvae (Fig. 1). Most protein spots with a molecular mass of 20000–90000 Da and pI of pH 3–10 were identified using MALDI-MS. NanoLC-MS was used to confirm protein identity by partial amino acid sequencing, if required.

### Classification of the identified hemolymph proteins of resistance-freezing and non-resistance-freezing ACBs

We analyzed the plasma proteome of resistance-freezing and non-resistance-freezing ACB larvae and identified 24 spots representing candidate parasitism-specific proteins. The peptide mass fingerprinting and the database search results were summarized in Table 1. The proteins identified in this study had diverse functional characteristics. We obtained the information from the SWISS PROT page, the Flybase, and from databases such as PROSITE or pfam^17^. Ms analysis revealed 21 protein spots after mass, including 17 upregulated and 4 downregulated in expression.

**Table 1:** Identification of the different protein spot between resistance-freezing and non-resistance-freezing ACBs.

Spot	Function	Protein name	Species	ACC. No	Related to cold response
A	Stress defense response	Heat shock protein hsp21.4	*B. mori*	GI|112983414	Verified in ACB^18^
B		Hsp70	*Trichoplusia ni*	GI| 1495233	Verified in ACB^19^
C		Mitochondrial stress-70 protein	*Monodelphis domestica*	GI|126290370	Verified in ACB^19^
D		Heat shock 70 kDa cognate protein	*Ostrinia furnacalis*	GI|324499424	Verified in *C. pipiens*^20^
E	Protein binding function	Actin	*Drosophila grimshawi*	GI|195036998	Verified in *C. pipiens*^20^
F		Actin1	*A. pernyi*	GI|460239208	Verified in *C. pipiens*^20^
G		Actin1 A3	*B. mori*	GI| 5751	Verified in *C. pipiens*^20^
H		Actin	*Cydia pomonella*	GI|46371991	Verified in *C, pipiens*^20^
I	Metabolism	Hydroxypyruvate isomerase	*B. mori*	GI|114052328	Not verified
J		S-formylglutathione hydrolase	*Danaus plexippus*	GI|357606390	Verified in *B. mori*^21^
K		Enolase	*B. mori*	GI|148298800	Verified in *A. pernyi*^22^
L		Arginine kinase	*B. mori*	GI|112983926	Verified in ACB^19^
M		Prophenoloxidase-2	*B. mori*	GI|9957279	Verified in *F. auricularia*^23^
N		Malate dehydrogenase 2, partial	*Papilio xuthus*	GI|389613549	Verified in *B.mori*^21^
O		Putative aldo-ketose reductase 1	*D. plexippus*	GI|357608136	Not verified
P		Putative serine protease-like protein 2	*D. plexippus*	GI|357615945	Verified in ACB^7^
Q		Chitinase-like protein EN03 precursor	*B. mori*	GI|112983920	Verified in *M.punctipennis*^24^
R	Ion regulation	Voltage-dependent anion-selective channel	*Helicoverpa armigera*	GI|328670887	Verified in *M. sativa*^25^ and *N. crassa*^26^
S	Gene expression	Eukaryotic translation initiation factor 3 subunit	*B. mori*	GI|114051800	Verified in *L. decemlineata*^27^
T	Immunity and nervous system	Yellow 2	*Biston robustum*	GI|294846071	Not verified
U		Putative lachesin	*D. plexippus*	GI|357619801	Not verified

The identified spots contained proteins that were already purified or characterized. Table 2 showed the protein identification data including the matched peptides and MASCOT score. Protein scores are derived from ions scores as a non-probabilistic basis for ranking protein hits. Individual ions scores > 53 indicate identity or extensive homology (*p* < 0.05). The MS proteomics data were deposited to the ProteomeXchange Consortium via the PRIDE^28^partner repository with the dataset identifier PXD044356. The datasets generated and/or analyzed in this study are also available in ProteomeXchange with the dataset identifier PXD044356.

**Table 2:** Matched peptides and MASCOT score of the different protein spot between resistance-freezing and non-resistance-freezing ACBs.

Spot	Matched peptides	MASCOT score
A	RFDVSQYTPEEIVVKT KLGDFSVIDTEFSSIRE RQLAEPSHWDSLNSPLIQDEGDGKT	251
B	KFELTGIPPAPRGKDAGTISGLNVLRIKSTAGDTHLGGEDFDNRMKNQVAMNPNNTIFDAKRLKTVQNAVITVPAYFNDSQRQ	287
C	KDAGQISGLNVLRV KVQQTVQELFGRA KNAVITVPAYFNDSQRQ	287
D	KFELTGIPPAPRGKDAGTISGLNVLRIKSTAGDTHLGGEDFDNRMKNQVAMNPNNTIFDAKRLKTVQNAVITVPAYFNDSQRQ	287
E	RAVFPSIVGRPRHKIWHHTFYNELRVKIWHHTFYNELRV	158
F	RAVFPSIVGRPRHKIWHHTFYNELRVKIWHHTFYNELRV	158
G	RAVFPSIVGRPRHKIWHHTFYNELRVKIWHHTFYNELRV	157
H	RGYSFTTTAERERAVFPSIVGRPRHKIWHHTFYNELRVKIWHHTFYNELRVRVAPEEHPVLLTEAPLNPKA	313
I	KYFLSDYGRAKLLPYIGHVQIAQVPNRN	118
J	KQLLPENLVEACRSKSVSAFAPICNPSACPWGVKA	93
K	RAAVPSGASTGVHEALELRDRGNPTVEVDLVTELGLFRAKVNQIGSVTESIDAHLLAKK	267
L	MVDAATLEKLEAGFSKLKETQQQLIDDHFLFKERGTRGEHTEAEGGVYDISNKR	252
M	RNVPWIFSDQRKRNVPWIFSDQRKMRGLDFSDNGPVYARFRFVTVLNAGENNIVRQRAAEGLFVTIDEMERWRQSTESSITIPFEQTFRD	483
N	KVLGVTTLDVVRARDDLFNTNASIVRD	157
O	KIDYIETWRGRHLDTAHLYRT	105
P	KANYLGYINKLDLSGKG	83
Q	RGLCTGDKYPILRAKEADYTAPIYTPQNRN	89
R	KEADYTAPIYTPQNRNKSLIGLGYQQKLMAPPYYADLGKKAKVTLEGTFAPQTGTKTKLRPGVTLTISAAIDGQNFNAGGHKV	378
S	KSYASGGEDGYVRVKLFDTNSLELLKEMKPLMLQGHQRARFFHLVFEEEFGRVKAMGHPCEVFVIDTRTRVHNFDQSYFDYTFDYNRE	525
T	RLFVTIPRRKVGNRLFVTIPRR	109
U	RISHFATADEFTDTTLRV	139

Some of the newly identified proteins in the hemolymph had their functional roles predicted based on specific homology domains shared with proteins identified in other species. All protein spots (except protein spot I, O, T, U) were verified related to cold response according to the previous research (Table 1). Proteins were classified into six functional groups: stress, protein binding, metabolism, ion regulation, gene expression, and nervous system.

### Proteins involved in stress-defense response and protein binding function

**Figure 2:**
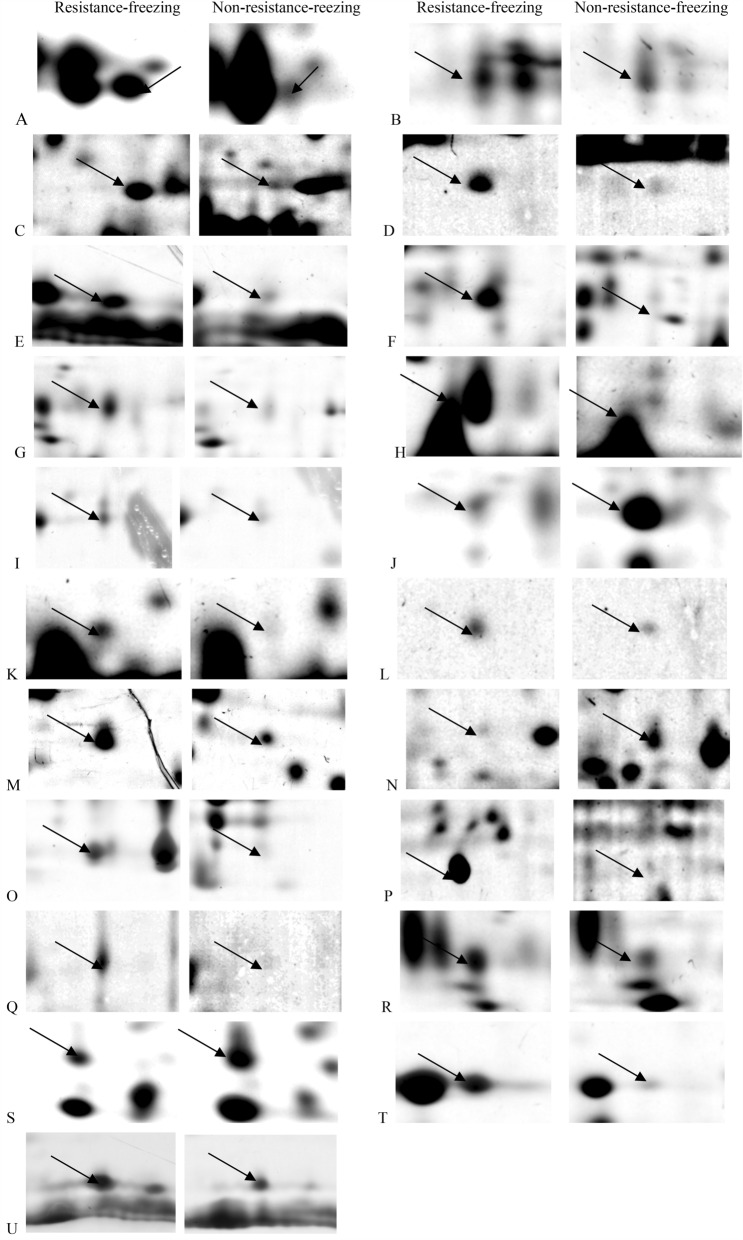
Partial maps of hemolymph proteins from resistance-freezing and non-resistance freezing ACB larvae. Three spots were expressed at the highest level in non-resistance freezing ACB larvae (J, N, S); the other proteins were highest in resistance-freezing ACB larvae (A, B, C, D, E, F, G, H, I, K, L, M, O, P, Q, R, T, U).

Four proteins may be crucial in responding to stress: heat shock protein (Hsp) 21.4 (Fig. 2A), Hsp70 (Fig. 2B), Heat shock 70 kDa cognate protein (Fig. 2D) and mitochondrial stress-70 protein (Fig. 2C). Hsps were discovered in a diverse array of insects, spanning the orders Lepidoptera, Diptera, Hymenoptera, Psocoptera and Hemiptera^29^. The well-characterized molecular responses to thermal extremes in insects remain those involving Hsps. Many Hsps are reported for essentially in environmental stress tolerance and thermal adaptation^30,31^, and often represent as sensitive indicators for phenotypic adjustments^32^. Our results indicated that these proteins exhibited an increase in spot density or volume after inducing resistance-freezing capacity.

Three constitutive proteins, actin (Fig. 2E, H), actin1 (Fig. 2F) and actin1 A3 (Fig. 2G), may play a role in protein binding functions. Actin proteins characterize the external coating of insects and form a complex with chitin, which is the main constituent of the exoskeleton^33^. Actin remodeling could form filopodial or lamellipodial cytoplasmic extensions, and actin-associated factors might act downstream of the hormone signaling pathway affecting immune responses in insects^34^. The members of this group exhibited an increase in spot density or volume after being induced with resistance-freezing capacity.

### Proteins involved in metabolism

Insect metabolism is affected by environmental, behavioral, developmental, and evolutionary factors. Temperature is an essential factor affecting insect metabolism. Nine proteins related to metabolism were identified as follows: hydroxypyruvate isomerase (Fig. 2I), S-formylglutathione hydrolase (Fig. 2J), enolase (Fig. 2K), arginine kinase (Fig. 2L), prophenoloxidase-2 (Fig. 2M), partial malate dehydrogenase 2 (Fig. 2N), putative aldo-ketose reductase 1 (Fig. 2O), putative serine protease-like protein 2 (Fig. 2P), and chitinase-like protein EN03 precursor (Fig. 2Q). Except S-formylglutathione hydrolase and partial malate dehydrogenase 2, the other proteins exhibited an increase in spot density or volume after being induced with resistance-freezing capacity.

### Proteins involved in ion regulation, gene expression, and nervous system

One protein, a voltage-dependent anion-selective channel, was related to ion regulation. This protein increased in spot density or volume after being induced with resistance-freezing capacity (Fig. 2R).

Eukaryotic translation initiation factor 3 subunit, involved in gene expression, decreased in spot density or volume after being induced with resistance-freezing capacity (Fig. 2S).

The yellow 2 and lachesin proteins were related to immunity and the nervous system. They increased in spot density or volume after being induced with resistance-freezing capacity (Fig. 2T, U).

## Discussion

ACB is a pest that damages cereal crops, including corn, sorghum, and millet^35^. This pest thrives well in tropical and subtropical environments but has developed certain resistance mechanisms to survive in cold environments. We investigated the differential expression of hemolymph proteins using 2DE. Different spots were identified in the protein maps of both strains. We observed a remarkable difference in the number of spots, especially in the resistant-freezing strain where this density or volume was significantly higher than that in the non-resistant strain.

Hsps are a group of highly conserved proteins in eukaryotic and prokaryotic cells. They are involved in a wide range of cellular processes, such as assisting protein folding and degradation of misfolded proteins, intracellular trafficking, modulating signaling pathways and regulating immune responses^36–40^. In this study, we identified four Hsp proteins (Hsp21.4, Hsp70, Heat shock 70 kDa cognate protein and mitochondrial stress-70 protein) related to stress-defense response. The spot density or volume of the identified proteins was different between the freezing and resistance-freezing ACB strains.

In insects, Hsps were expressed constitutively and induced by environmental stresses such as heat and cold^41^. Hsp70 plays a crucial role in various folding processes, including the folding and assembly of newly synthesized proteins, the refolding of misfolded and aggregated proteins, the translocation of organellar and secretory proteins across membranes, and regulation of the activity of regulatory proteins^42,43^. Proteins such as Hsp70 can respond to a range of adverse conditions and also interact with numerous other proteins in producing phenotypic effects under stress^32,44,45^. The expression pattern of *Hsp70* gene is species- and breed- specific, probably because of varying degrees of thermal tolerance and acclimatization to seasonal variations^46^. Hsp70 plays a crucial role not only in the development of rapid cold hardiness but also in cold stress recovery. This was corroborated by the significant induction of *Hsp70* to cold response with different temporal expression patterns in *Bemisia tabaci* and *Dermacentor silvarum*^47,48^. The expression of Hsp70 and other Hsps in insects can exert indirect effects on plastic responses by impacting the development of stress-resistant diapause phases, although these effects seem to be specific to particular taxonomic groups^49–53^. Genes from four Hsp families showed variable expression levels among tissues and developmental stages in *Grapholita molesta*, and *Hsp21.4* displayed the highest expression immediately after heat stress^18,54^.

Actin, which is present not only in the cytoplasm but also in the nuclei of somatic cells and oocytes^55^, is a multifunctional protein that functions after the creation of a microfilament with other proteins. Actin is involved, along with myosin, tropomyosin and troponin, in forming muscular myofibrils that play a crucial role in muscle contraction^56^. Both myosin and tropomyosin belong to the category of muscle proteins. In diapausing insects, such as *O. furnacalis* and *Culex pipiens*, cold tolerance and prolonged lifespan are important features crucial for overwintering success. RNA interference-aided knockdown of *PDZ* gene significantly decreased actin accumulation in the midgut of early-stage adult diapausing female mosquitoes^20^. Our results revealed that four actin proteins were identified and exhibited an increase in spot density or volume after being induced with resistance-freezing capacity. The results revealed that the temperature is an actin-associated factor and might affect actin remodeling.

Seven proteins (hydroxypyruvate isomerase, enolase, arginine kinase, prophenoloxidase-2, putative aldo-ketose reductase 1, putative serine protease-like protein 2 and chitinase-like protein EN03 precursor) exhibited an increase in spot density or volume after being induced with resistance-freezing capacity. Hydroxypyruvate isomerase is found widely in prokaryotes to eukaryotes, suggesting that the enzyme reaction or the enzyme itself was physiologically important. The hydroxypyruvate isomerase gene was cloned and characterized in *Bombyx mori*; however, whether the glyoxylate cycles existed in insects was not indicated^57^. Hydroxypyruvate isomerase, which increases in resistance-freezing ACB, may be crucial in understanding the role of the protein in insects. Enolase is an important glycolytic enzyme found in all organisms. Glycolysis plays an important role in energy production across all organisms^58^. In recent years, many insect enolases have been discovered, each having various species-specific roles^59,60^. In *Antheraea pernyi,* the expression of *enolase I* gene was significantly downregulated in response to cold shock and significantly upregulated upon to heat shock, suggesting that the gene was induced by temperature stress^22^. This study indicated that enolase was related to resistance-freezing capacity.

Arginine kinase plays an essential role in insect energy metabolism^61^. This protein was overexpressed in the overwintering of ACB larvae^19^. Chitinases are the key chitin degradation enzymes and are critical for regulating insect growth and development^62^. The chitinase genes from cold-treated desert beetle *Microdera punctipennis* had tissue-specific and cold-inducible expression, suggesting that these chitinases might have diverse functions and played roles in insect cold adaptation^24^. The prophenoloxidase activating system in insects plays a crucial role in defense against microbial invasion^63^, and serine proteases are a common functional category in insect genomes that can have diverse roles in parasitoid physiology^64^. The prophenoloxidase activity was 30% higher in female *Forficula auricularia* who underwent long-winter treatment compared with those undergoing short-winter treatment^23^. RNA-sequencing and quantitative real-time polymerase chain reaction analyses revealed that the expression of *serine protease* gene was upregulated in ACB larvae under cold stress at 8°C^7^. In this study, four proteins exhibited an increase in energy metabolism and immune capacity of ACB during the induction of resistance-freezing capacity.

S-formylglutathione hydrolase is the key rate-limiting enzyme in the carbon cycling pathway, and partial malate dehydrogenase 2 is involved in the tricarboxylic acid cycle and glycolysis^23^. The expression of *S-formylglutathione hydrolase* gene was downregulated significantly after adding AgNPs in *B. mori*^65^. Our results showed that S-formylglutathione hydrolase exhibited a decrease in spot density or volume after being induced with resistance-freezing capacity. This resulted in the slower use of carbohydrates by fat bodies and associated metabolic changes.

The voltage-dependent anion-selective channels are located in the mitochondrial outer membrane and are the major pathway for metabolism and ion transport across the mitochondrial outer membrane^66–68^. The correlation of cold and voltage-dependent anion-selective channels was analyzed in some plants (*Medicago sativa*)^25^ and microorganisms (*Neurospora crassa*)^26^. In resistance-freezing ACB, the voltage-dependent anion-selective channels exhibited an increase in spot density or volume. This finding suggested that mitochondrial metabolism and function changed during the induction of resistance-freezing capacity.

Protein synthesis in eukaryotic cells is regulated in response to growth factors, hormones, and changes in the external environment. The initiation of mRNA translation is a key control point for protein synthesis and is mediated by various eukaryotic initiation factors^69^. Eukaryotic translation initiation factors play a role in regulating the activity of prothoracic glands as signaling factors^70,71^. Elevated levels of multiple proteins, including the eukaryotic translation initiation factor associated with stress granule formation and translational regulation, were observed in cold-exposed *Leptinotarsa decemlineata*^27^. In ACB, we found that inducing resistance-freezing led to the downregulation of enzymes activity on eukaryotic translation initiation factors.

Lachesin is a cell surface protein expressed in neurogenic cells early in development, before morphological changes associated with neuronal differentiation are apparent. This protein also plays a role in early neuronal differentiation in axon outgrowth, cell recognition events, cell adhesion, and intercellular communication^72,73^. The upregulation of putative lachesin in resistance-freezing ACB revealed that the insect nervous system might have had some changes during induction.

To date, evolution theories have proposed that organisms exposed to adverse environmental conditions have to deal with a trade-off of energy and have limited resources for growth, reproduction, and defense to cope with these conditions^74^. Although this condition may be advantageous under certain circumstances, it also can simultaneously reduce several physical and chemical functions, as well as genetic mechanisms and energy assignment (antagonistic pleiotropy)^75,76^. Further, the diversity in the number of spots in protein maps (the freezing and resistance-freezing) suggests that many proteins are involved in the resistance-freezing induction.

Proteomics research helps investigate protein expression patterns, and identifies many attractive candidates for further investigations. In this study, we explored the role of hemolymph proteins in the freezing and resistance-freezing ACB strains using a proteomic approach. Ms analysis revealed that 21 protein spots were identified, including 17 upregulated and 4 downregulated in expression. These proteins play an essential role in metabolism, energy supply and defense reaction in insects, 17 protein spots were reported to be related to cold response. But only 5 protein spots (A, B, C, L, P) were verified related to cold response in ACB. Those protein spots, which were not been verified related to cold response in the current references but were found in this study, will be focus on in the next research work. We will examine various genes in the freezing and resistance-freezing ACBs, confirming the correlation between our results and these genes. Subsequently, we verified the functions of the upregulated and downregulated proteins through western blotting, mRNA, subcellar location and protein interaction in the future.

## Materials and methods

### Insects and sample preparation

The fifth instar larvae of ACB and the artificial diet were obtained from the Pest Biological Control Laboratory, Shenyang Agricultural University. The ACB larvae reared on an artificial diet (soybean meal, 13%; agar, 4%; rice bran, 16%; yeast, 6.2%; sorbic acid, 0.3%; methyl benzoate, 0.5%; tetracycline, 0.05%; vitamin mixture, 0.5%; and casein, 0.3%) at 27 °C ± 1 °C and 75% humidity over a 16 h/8 h light/dark photoperiod in a plastic cage (35 × 25 × 21 cm^3^). ACB overwinter in the final instar larva diapause stage, so the final instar larva stage is the optimal time to study the resistance-freezing^27^. The post-diapause female ACB larvae in treatment groups were exposed to resistance-freezing outdoor conditions, for inducing resistance to freezing from September 10th to October 31st, at the average temperature of − 5 °C to 10 °C. The control group consisted of post-diapause female ACB larvae that did not develop resistance-freezing capacity indoors. The ACBs were pricked and squeezed gently to collect hemolymph. Each sample was collected from approximately five ACBs as a biological replicate, and five experimental replicates were performed.

The samples were centrifuged at 40000 rpm and 4 °C for 30 min. Hemocytes and debris were discarded, and the supernatant was stored in a lysis solution containing 8 M urea, 4% (w/v) 3-[(3-cholamidopropyl)-dimethylammonio]-1-propane sulfonate (CHAPS), 1% (w/v) dithiothreitol (DTT), and 1% (v/v) protease inhibitor cocktail (Sigma P2714). The total protein content in the supernatant was determined using Bradford’s method^77^.

### 2D-PAGE and SDS-PAGE

For analytical 2D gels, about 200 μg of protein was solubilized in a rehydration solution (8.0 M urea, 2% CHAPS, 0.8% DTT, 0.5% IPG buffer, pH 3–10 and 0.002% bromophenol blue) and applied to an IPG strip (11 cm, linear pH 3–10) (Bio-Rad) using rehydration buffer (DeStreak Rehydration Solution, Bio-Rad). Rehydration was performed for 12 h at 20 °C. Isoelectric focusing (IEF) used a sequential gradient procedure of 250 V for 30 min, 1000 V for 30 min, 8000 V for 4 h, and 8000 V for 40, 000 h using the IPG-phor system (Bio-Rad). The current limit was 50 μA per IPGstrip. After IEF, the strips were incubated in equilibration buffer A (6 M urea, 50 mM Tris–HCl, 2% SDS, and 20% glycerol) containing 13 mM DTT for 15 min, and then alkylated for 15 min in equilibration buffer containing 2.5% iodoacetamide. The strips were loaded onto 12.5% polyacrylamide gels for the second-dimension separation using the SE 600 Vertical Gel Electrophoresis Unit^78^. SDS-PAGE was conducted at 5 mA per gel for 20 min, followed by 10 mA per gel until the bromophenol blue dye reached the end of the gel.

The gels were silver-stained to visualize protein spots^79^. The gels were stained with silver nitrate following the Amersham 2D handbook. They were then developed in a rinse solution for approximately 5 min, and subsequently transferred to a stop solution before the background turned dark^80^. Proteins exhibiting differential expression in the resistance-freezing and non- resistance freezing ACBs were excised from the 2D gels.

### Image and statistical analyses

The gel images were scanned with a high-resolution image scanner II (Amersham Bioscience, USA) set to 800 pixels, and analyzed using PD Quest software. The analysis included an automated spot detection and matching function, followed by manual designation of spots as landmarks for gel alignment. A master gel was constructed to summarize the spots detected consistently. During parasitism, spot intensities were normalized, and a quantitative analysis was performed. Protein spots that showed at least a twofold differential regulation in resistance-freezing and non-resistance-freezing ACB larvae were selected for further examination. The normalized volumes of these spots were subjected to statistical analysis using the *t*-test. All samples were run in triplicate, and only spots that replicated in at least two out of the three replicates were considered for analysis. A comparison was made between resistance-freezing and non-resistance-freezing and the differences in spot densities were identified.

### In-gel digestion, MALDI-TOF MS, and protein identification

Protein spots were excised from the gel with a scalpel, and destained with 100 μL of 25 mM NH_4_HCO_3_ and 50% Acetonitrile (ACN), with occasional vortexing until the brown color disappeared completely. The gel pieces were rinsed three times with Milli-Q water. The supernatant was discarded, and the gel pieces were dehydrated with 50% ACN for 30 min and 100% ACN for 30 min. The pieces were then rehydrated with 15 μL of 25 mM ammonium bicarbonate containing 10% ACN and, 0.3 μg of modified trypsin (Sigma Aldrich, USA). Following overnight digestion at 37 °C, the peptides were extracted with 25 μL of 67% ACN containing 5% trifluoroacetic acid (TFA). The supernatant was transferred into a new tube and completely dried. The combined extraction was resuspended in 5 μL of 0.1% TFA, mixed in a 1:1 ratio with a matrix consisting of a saturated solution of α-cyano-4-hydroxy-trans-cinnamic acid in 50% CAN and 0.1% TFA. A 1μL aliquot of the peptide mixture was spotted on a stainless-steel sample target plate. Peptide MS and MS/ MS analyses were conducted using an Ultraflex TOF/ TOF (Bruker, Germany) instrument. The analyses were performed in positive MS reflector mode, covering a mass range of 750–3000 Da. Both the MS and MS/ MS data were integrated using Mascot2.3 (Matrix Science Ltd., London, U.K.) for protein identification. Proteins were successfully identified based on combined MS and MS/ MS spectra, using a confidence interval of 95% or higher in the MASCOT V2.3 search engine (Matrix Science). The search parameters included the NCBInr-Animals database, trypsin as the digestion enzyme, allowance for one missed cleavage site, and fixed modifications of carbamidomethyl (C). Partial modifications considered were Acetyl (Protein N-term), Deamidated (NQ), Dioxidation (W), and Oxidation (M). The search also incorporated a precursor ion tolerance of 100 ppm and a fragment ion tolerance of 0.3 Da.

To identify the proteins, all MS/ MS spectra recorded for tryptic peptides derived from the spots were searched against protein sequences from NCBInr databases using the MASCOT search program (www.matrixscience.com, Matrix Science) with MS-BLAST^81^. The parameters for all searches included a maximum of one allowed missed cleavage, a mass tolerance of 1 Da, and deisotoped masses of peaks for protein identification. The fixed covalent modifications considered in the search procedure is C, and potential covalent modifications included ProteinN-term, NQ, W and M.

## Data Availability

The datasets used and/or analyzed during the current study available from the corresponding author on reasonable request.
